# Protective effect of citronellol in rhabdomyolysis-induced acute kidney injury in mice

**DOI:** 10.25122/jml-2023-0102

**Published:** 2023-07

**Authors:** Yasameen Shakir Mahmood, Sarmed Hashim Kathem

**Affiliations:** 1 Ministry of Health and Environment, Karbala Health Directorate, Karbala, Iraq; 2 Department of Pharmacology and Toxicology, College of Pharmacy, University of Baghdad, Baghdad, Iraq

**Keywords:** Rhabdomyolysis, AKI, BAX, Cleaved caspase-3, anti-apoptotic effects

## Abstract

Acute kidney injury (AKI) is a serious pathophysiological event consequent to rhabdomyolysis. Inflammatory mechanisms play a role in the development of rhabdomyolysis-induced AKI. Citronellol (CT) is a naturally occurring monoterpene in essential oils of aromatic plant species. In this study, we explored the protective effects of citronellol on AKI resulting from glycerol-induced rhabdomyolysis. Rhabdomyolysis was induced by a single intramuscular injection of glycerol 50% (10mg/kg) in the thigh caudal muscle. Four groups of mice were assigned, including a control group, a group administered with glycerol to induce AKI as a model, a group treated with glycerol plus 50mg/kg CT, and a group treated with glycerol plus 100mg/kg CT. The renal function of mice from all groups was evaluated using kidney histopathological changes and kidney injury molecule-1 (KIM-1). Myoglobin levels were measured to detect rhabdomyolysis. Apoptosis was evaluated by renal cleaved caspase-3 and BAX levels. Both doses of citronellol (50mg/kg and 100mg/kg) significantly reduced KIM-1 mRNA expression and myoglobin levels compared to the glycerol group. In addition, citronellol resulted in lower cleaved caspase-3 and BAX in the renal tissue, indicating that citronellol exerted an anti-apoptotic effect in AKI. Citronellol showed a reno-protective effect against rhabdomyolysis-induced AKI, which may be attributed to its anti-apoptotic effects.

## INTRODUCTION

Rhabdomyolysis is a serious medical condition that occurs when the muscle tissue is injured and releases its contents into the bloodstream. This can happen due to various causes, including trauma, infections, drug use, and extreme exercise [1]. Acute kidney injury (AKI) is a major health complication resulting in end-stage renal disease (ESRD) and even death if not promptly diagnosed and treated [2]. Myoglobin is a protein freely filtered by the glomeruli in the kidneys and then reabsorbed by the proximal tubules. When there is an excessive release of myoglobin into the bloodstream, such as in cases of rhabdomyolysis, it can lead to the accumulation of myoglobin in the tubular cells of the kidneys. This can cause oxidative stress, inflammation, and cell death, producing AKI [3]. Caspase-3, a vital apoptosis executor, and Bcl-2-associated X protein (BAX), a proapoptotic protein that stimulates cytochrome c release from mitochondria, are implicated. Activating caspase 3 and subsequent cell apoptosis in the kidneys can contribute to developing and progressing rhabdomyolysis-induced AKI [4]. Glycerol injection is commonly used to induce rhabdomyolysis in animal models [4]. Citronellol (CT) is a naturally occurring monoterpene alcohol (3,7-Dimethyl-6-often-1-ol) found in some species such as Cymbopogon citrates, Cymbopogon wineries, and Lippiaa alba and commonly used as a fragrance and flavoring agent. It is also used in various industries, including the food, cosmetic, and pharmaceutical industries. Citronellol exists in two forms: (+)-CT, primarily in citronella, Amyris, and eucalyptus citriodora oils, and (-)-CT. It is mostly present in pelargonium and rose oils (both are colorless liquids with a rose fragrance). Citronellol has been shown to have antibacterial, antifungal, and repellent properties in vitro and cardiovascular, antidiabetic, and antinociceptive properties in vivo [5]. There is currently no medication available to treat or prevent AKI or rhabdomyolysis [6, 7]. In our study, we investigated the anti-apoptotic effect of citronellol in a mouse model.

## MATERIAL AND METHODS

### Animal

The College of Pharmacy at the University of Baghdad provided the mice used in our experiments. All procedures performed on the animals strictly adhered to both institutional and international regulations governing the ethical treatment and use of laboratory animals.

### Experimental protocol

The study used 32 male BALB/c Albino mice weighing 25-32 gr. The mice had free access to water and a standard diet. Rhabdomyolysis was induced in the mice by subjecting them to 24 hours of water deprivation, followed by a single intramuscular injection of glycerol 50% (obtained from Merk, Germany) at a dose of 10 ml/kg [2, 8]. The mice were randomly divided into four groups, each consisting of 8 mice. The control group received normal saline for 4 consecutive days. The model group received a single intramuscular injection of glycerol 50% at a dose of 10 mg/kg to induce rhabdomyolysis. The treatment groups were administered citronellol (obtained from Sigma-Aldrich, Germany) orally at doses of 50 mg/kg and 100 mg/kg for 4 days, followed by a single intramuscular injection of glycerol 50% at a dose of 10 mg/kg on the fourth day [6]. All mice were euthanized 24hrs after glycerol injection by diethyl ether followed by cervical dislocation [8]. The right kidneys were extracted and homogenized [9, 10] and kept frozen for later measurement of myoglobin, cleaved caspase-3, and BAX using ELISA (MY BioSource, USA). The left kidneys were fixed in formalin for histopathological analysis using hematoxylin and eosin (H&E) staining [9, 10].

### Gene expression analysis

Gene expression analysis of KIM-1 was conducted by measuring mRNA levels in kidney tissue using a standard quantitative reverse transcription-polymerase chain reaction (qRT-PCR) protocol [11]. In summary, kidney homogenate with TRIzol was used to isolate total RNA using TransZol Up Plus RNA Kit (TransGen, biotech). Subsequently, complementary DNA (cDNA) synthesis was performed using the EasyScript^®^ one-step gDNA removal and cDNA synthesis (TransGen, biotech). SYBR Green Supermix (TransGen, biotech) performed the mRNA expression levels with GAPDH as a housekeeping gene. The primer sequences were forward GAPDH CGGGTTCCTATAAATACGGACTG and reverse CCAATACGGCCAAATCCGTTC; KIM-1 forward, GGCTCTCTCCTAACTGGTCA and reverse TGATGTGCTGCTGCGAGATT.

## RESULTS

### Effect of citronellol on kidney function and injury

**Figure 1:**
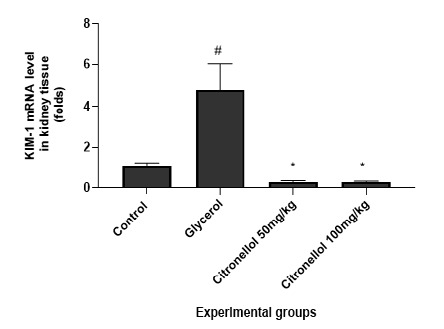
KIM-1 expression in renal tissue. The results are expressed as mean±SEM, and statistical significance was determined by comparing the control group with the glycerol-injected mice and the citronellol-treated mice with the glycerol-injected mice. A p-value of <0.05 (#p<0.05 or *p<0.05) indicates statistical significance.

**Figure 2:**
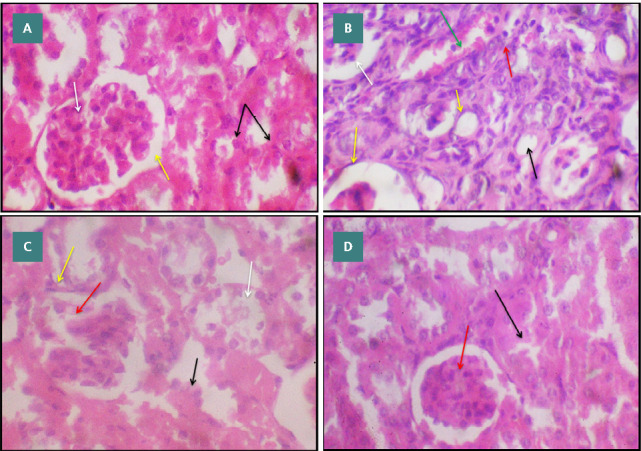
A: Photomicrograph of kidney section of control mice showing the normal histological architecture of the renal cortex and normal glomerular tuft morphology (white arrow). Significant and regular epithelium of renal tubules (black arrow) with definite renal corpuscle (yellow arrow). (H and E,40X). B: Photomicrograph of kidney section of glycerol-induced renal injury showing significant histopathological alterations, severe glomerular atrophy (white arrow), significant dilatation of renal tubules lumen (black arrow), with remarkable periglomerular and peritubular fibroblast infiltration (yellow arrow), massive polymorphonuclear cells interstitial infiltration (red arrow) and severe congestion of blood vessels (green arrow). (H and E,40X). C: Photomicrograph of kidney section of citronellol 50 mg\kg treated mice showing slight histological reversible alterations, mild glomerular necrotic changes (red arrow), degeneration of tubular endothelial (black arrow), with endothelial necrosis and desquamation (white arrow), and peri corpuscular inflammatory cells infiltration (yellow arrow). (H and E,40X). D: Photomicrograph of renal cortex section of a citronellol 100 mg\kg treated mice showing significant histological reversible alterations, close to normal glomerular morphology (red arrow), mild degeneration of tubular endothelial represented by a star-shaped lumen (black arrow). (H and E,40X). E: semi-quantitative scoring system of kidney injury.

To assess renal function, kidney KIM-1 expression and histopathological evaluation of the kidney were used. Glycerol injection in mice is known to cause deterioration in renal function, which is reflected by a significant elevation in KIM-1 expression (4.82±1.21 *vs*. 1.08±0.16) compared to control, as shown in Figure 1. Data revealed that pre-treatment with CT 50&100mg/kg resulted in significant downregulation in KIM-1 expression (0.29±0.07 & 0.29±0.04 *vs*. 4.82±1.21), respectively, compared to the model group. In addition, the histological evaluation of kidney tissue further supported these results. Kidney injury was evaluated by a semi-quantitative scoring system of kidney injury, which was estimated for each animal observed in a blinded manner. The percentage of tubules in the renal cortex that showed significant histopathological alterations, severe glomerular atrophy, significant dilatation of renal tubules lumen, with remarkable periglomerular and peritubular fibroblast infiltration, massive polymorphonuclear cells interstitial infiltration, and severe congestion of blood vessels, were scored as follows: 0, normal kidney; 1, <25%; 2, 25–50%; 3, 50–75%; 4, >75% [12]. As shown in Figure 2 (A-D), CT treatment exhibited a dose-dependent improvement in kidney injury scores compared to the model group.

### Effect of citronellol on myoglobin

**Figure 3:**
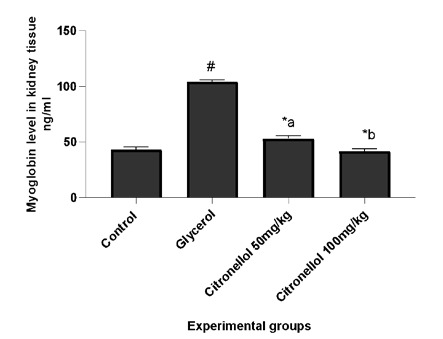
Results of an experiment conducted on albino mice to investigate the effect of citronellol on myoglobin in kidney tissue

Myoglobin was measured to assess rhabdomyolysis. Injection of glycerol resulted in spike elevation of myoglobin (104.02±1.91 *vs*. 43.09±2.41) levels compared to control, as shown in Figure 3. Interestingly, mice receiving CT 50&100mg/kg for 4 days revealed a significant dose-dependent reduction in myoglobin (53.18±2.56 & 41.78±2.05 *vs*. 104.02±1.91) compared to the model group, implying an improving effect on muscle rhabdomyolysis. The parameter was measured 24 hours after glycerol injection. The results are expressed as mean±SEM, and statistical significance was determined by comparing the control group with the glycerol-injected mice and the citronellol-treated mice with the glycerol-injected mice. A p-value of <0.05 (#p<0.05 or *p<0.05) indicates statistical significance and a and b referred to treatment groups.

### Effect of citronellol on apoptosis of tubular epithelial cells

**Figure 4:**
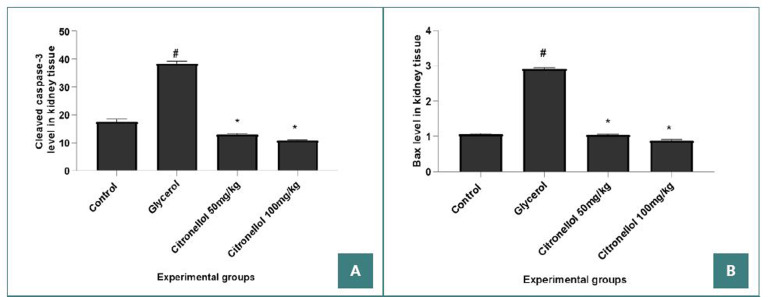
Cleaved caspase-3 and BAX levels in kidney tissues. (A) Cleaved caspase-3 (B) BAX level.

In this study, we evaluated the effect of CT on the levels of two markers of apoptosis, cleaved caspase-3 and BAX. Intramuscular injection of glycerol resulted in significant elevation of kidney tissue cleaved caspase-3 (#) and BAX (#) compared to control (38.30±0.87 *vs*. 17.39±1.075) and (2.90±0.03 *vs*. 1.05±0.01) respectively, indicating amplified renal apoptosis due to rhabdomyolysis as shown in (Figure 4 A, B). Remarkably, mice treated with either CT 50 mg/Kg or 100 mg/Kg showed a significant decline in both apoptosis markers cleaved caspase-3 (12.84±0.37 & 10.68±0.43 *vs*. 38.30±0.87) and BAX (1.03±0.01 &0.87±0.038 *vs* 2.90±0.03) compared to model group (Figure 4 A, B). This effect revealed that CT exerted a reno-protective effect through anti-apoptotic action in this mouse model.

## DISCUSSION

Acute kidney injury (AKI) is associated with serious rhabdomyolysis. Glycerol-induced AKI is a commonly used experimental model for studying the mechanisms underlying AKI and developing new approaches for its treatment [12, 13]. In this model, glycerol is injected into the muscle, which causes the breakdown of muscle cells and the release of myoglobin, creatine kinase, and other toxic substances into the bloodstream. These substances can overwhelm the kidney's filtering capacity, damaging the renal tubules and impairing kidney function [13-15]. Based on the results of this study, it appears that glycerol injection induced rhabdomyolysis in the tested groups, as evidenced by the significant increase in Myoglobin concentrations (as observed in previous studies [16]) compared to the control group. Additionally, the glycerol group showed significant kidney damage and morphological changes in kidney tissue [17, 18].

However, the administration of citronellol at both 50 and 100 mg/kg doses appeared to have a protective effect on the kidneys of the tested animals. The treatment significantly reduced renal tubular epithelial cell degeneration and necrosis. The observed reduction in tubule necrosis scores and morphological damage suggests that citronellol may play a role in preventing or treating rhabdomyolysis-induced kidney injury.

KIM-1 is a protein that is upregulated in response to kidney injury and is primarily expressed in the kidney's proximal tubules. KIM-1 is a promising biomarker for the early detection of AKI [19]. Studies have shown that KIM-1 expression in the kidneys greatly increases in response to injury or insult, such as ischemia or exposure to nephrotoxic agents [20]. The results showed that KIM-1 expression increased after glycerol injection [21], and both doses of citronellol led to a significant decrease in KIM-1 gene expression compared to the glycerol group. This suggests that citronellol may protect against the injury caused by glycerol.

The toxic effects of myoglobin and other substances on the kidney are mediated by oxidative stress and inflammation. One of the consequences of rhabdomyolysis is the accumulation of lipid peroxides, which are reactive molecules that can cause damage to cell membranes and other cellular components [22]. Reactive oxygen species (ROS) can be produced when the electron transport chain is interrupted by damage to the mitochondrial membrane. Highly reactive chemicals known as ROS have the potential to further harm biological components such as DNA, proteins, and lipids [22]. Cytochrome C may also be released into the cytoplasm due to mitochondrial membrane breakdown [23]. This event triggers the activation of caspases 1 and 3, enzymes that play a key role in programmed cell death or apoptosis [24]. Tubular-cell apoptosis is a common feature of rhabdomyolysis-induced acute kidney injury [25, 26].

Caspases are enzymes that are crucial in programmed cell death or apoptosis. They are important apoptotic pathways and are triggered in response to various stimuli, including inflammation, oxidative stress, and other variables. The Bcl-2 family proteins, such as BH3 interacting domain death agonist (BID) and Bcl-2-associated death promoter (Bad), are activated by various stimuli in the mitochondrial pathway of apoptosis. Upon activation, Bcl-2-associated X (BAX) and Bcl-2 antagonist/killer (BAK) is triggered, releasing cytochrome c (Cyt c) from the mitochondria. Extracellular ligands, including tumor necrosis factor and Fas, attach to the extracellular receptors, triggering caspase 8. Then, after cleaving and activating caspase-3, apoptosis is started [27-29].

In this study, we found that cleaved caspase-3 and BAX were increased in the kidneys of mice in the glycerol group, as observed in another study [29, 30] but significantly decreased in the mice treated with citronellol in both low and high doses. This suggests that treatment with citronellol may reduce apoptosis in the kidneys of mice.

## CONCLUSION

The study demonstrated that citronellol has a reno-protective effect against rhabdomyolysis-induced acute kidney injury in mice, which may be attributed to its anti-apoptotic effect. The findings suggest that citronellol may be a promising therapeutic agent for AKI. The results of this study provide valuable insights for future research and the development of effective treatment strategies for AKI. However, further studies are needed to validate the efficacy and safety of citronellol in human subjects.
